# The Care of the Vulcanizer

**Published:** 1890-07

**Authors:** George B. Snow

**Affiliations:** Buffalo, N. Y.


ARTICLE IV.

### THE CARE OF THE VULCANIZER.

BY GEORGE B. SNOW, D. D. S., BUFFALO, N. Y.

The occasional reports of explosions of dental vulcanizers, accompanied as they are by more or less damage to property, and imminent risk of personal injury, renders the question of their avoidance one of considerable interest. Their entire prevention is an impossibility, so long as the management of the vulcanizer is entrusted to boys, or persons who know nothing and care still less about the properties of steam, or the rules upon the observation of which depend the safety and proper operation of the machine committed to their charge. It is seldom, if ever, that inquiry into the circumstances attending a vulcanizer explosion fails to elicit the fact that it was the consequence of gross carelessness or ignorance. The safety apparatus is often deliberately put out of order, and all the chances of an accident which may happen from a few moments' forgetfulness are taken by the operator, who probably knows about as much of the properties of steam as a Christian scientist does of human physiology.

It is proposed, in the present article, to touch upon a few points in the matter of the proper care of vulcanizers, and more especially to show how and when they become unsafe by use. It seems to be the opinion of some dentists that a vulcanizer should remain good indefinitely; but it is often the case that one returned to the maker for supposed trivial repairs, is found to be in such condition that its further use would be attended with great risk.

Vulcanizers, as they leave the manufacturer's hands, may be depended upon as strong enough to withstand a pressure three or four times as great as that incident to the vulcanizing process. No house, having any care for its reputation, can afford to put out one unless it is of undoubted strength. They are also provided with ample safeguards, by means of which they will be relieved from over-pressure, if it should occur. But no such appliance can be made which cannot be—either ignorantly or designedly—put out of order; neither can a vulcanizer be made so strong that it will be safe under any attainable pressure. It must be carefully and intelligently managed to insure safety.

Vulcanizers are gradually weakened and eventually destroyed by corrosion. The original thickness of the metal is fully preserved at the bottom of the pot, and there is but slight deficiency at the mouth. The action is almost wholly upon the sides, and at the middle the metal wastes away until it is scarcely thicker than heavy paper. Still a vulcanizer was used until it had reached this state of deterioration, without giving way. This shows how little strength is really required to withstand the pressure due to the vulcanizing temperature, if care is taken that it be not exceeded.

When the sides of the vulcanizer are weakened by corrosion to any great extent, the fact is easily ascertained by tapping them lightly with a small hammer. If the metal is thick and strong, it will be elastic, and the hammer will rebound from a light blow, though of course copper would yield to a heavy one. When the metal is quite thin, the sensation will be as though the blow were delivered upon lead. There will be little if any rebound, and the metal will be driven in and dented by a very light blow.

Corrosion occurs to a certain extent from exposure of the vulcanizer to air and moisture. Indeed, it is by no means sure that the greater part of it does not thus take place. It is good practice to clean the vulcanizer pot, and wipe it dry before laying it away after use.

Another cause of failure in vulcanizers, one which happily occurs but seldom, is the cracking of the metal near the corner of the bottom. As the bottom is usually covered with scale, it may crack and even give way before the existence of any defect is suspected. This fact forms another argument for keeping the vulcanizer clean, as before mentioned.

When a screw fastening like that of the Whitney vulcanizer is employed, mischief is often done by the inordinate use of black-lead or soap-stone powder upon the packing point, and incidentally upon the screw. The particles of which either of these powders are composed, are hard enough to wear away metal if placed between two rubbing surfaces, and in consequence the screw threads of vulcanizers are sometimes so worn that they have not sufficient hold upon each other to retain the cover; which on some fine day mounts to the ceiling, and disappears in the lath and plaster, much to the surprise and disgust of the owner.

The reason for applying soap-stone or plumbago powder to the surface of the packing seems, in many cases, to be entirely misapprehended. Its only office is to prevent the packing from sticking to the edge of the pot. A minute quantity only is required for this purpose, and its application need be made but seldom. If it is applied too liberally or too often, it will form a thick coating on the surface of the packing, which will be porous and will be the cause of leakage. When the coating attains any great thickness, it will scale off; and the leakage, which may have been almost imperceptible before, will now be increased to such an extent as to be annoying. Possibly the dentist does not detect the cause of the trouble, and, thinking that the vulcanizer "works hard," applies oil to the thread. This is burnt by the heat of vulcanizing, and the cover is virtually cemented to the pot. It is now removed with difficulty, if at all. As a rule, when the packing of a vulcanizer is in good order and steam-tight, the less that is done to it the better.

There is another matter connected with the management of the vulcanizer, which frequently causes great annoyance if not properly understood. If the vulcanizer is too full of water, not allowing adequate room for its expansion when heated, a pressure will be developed much greater than that due to the production of steam. The safety disk will in this instance be blown out, possibly at as low a temperature as 280° or 300°. Or if the safety apparatus be put out of order, the vulcanizer pot may be bulged and stretched out of shape, or a rupture may be the result, and a so-called explosion ensue.

It must be remembered that water is inelastic, and that when it is confined, with inadequate room for its expansion, the resulting force is practically irresistible. It is an easy matter, if the vulcanizer be wholly filled with water, to obtain a pressure of six, eight, or nine hundred pounds to the square inch, without heating the water to the boiling point. Is it any wonder that they sometimes give way when carelessly used?

A safe rule is to allow one-sixth at least, better one-fourth, of the capacity of the vulcanizer for steam room. The user of a vulcanizer should never lose sight of the fact that it is a steam boiler, and is subject to deterioration by use. The rules for its management should be thoroughly read till clearly understood, and carefully observed, and after the vulcanizer has been in use for a time it should be inspected frequently, and any signs of weakness carefully noted.—*Dental Advertiser.*

